# Falciform ligament use in perforated duodenal ulcer repair: a case report and literature review

**DOI:** 10.1093/jscr/rjaa243

**Published:** 2020-11-12

**Authors:** Amr Elgazar, Ahmed K Awad, Sheref A Elseidy

**Affiliations:** Department of General Surgery, Ain Shams University Hospitals, Cairo, Egypt; Department of Cardiovascular Diseases, Ain Shams University, Cairo, Egypt; Department of Cardiovascular Diseases, Ain Shams University, Cairo, Egypt

**Keywords:** duodenal ulcers, perforation, omental patch, falciform ligament, case reports

## Abstract

Acute perforated duodenal ulcers are considered one of the most encountered emergency surgical conditions leading to mortality. Different approaches have been proposed for management based on the clinical status of the patient. The use of omental patch closure is widely accepted either via an open or laparoscopic approach. However, not all patients are candidates owing to the availability and viability of the greater omentum. In these patients, the falciform ligament can be used as an alternative and efficient method for repair. In this case, we present a male patient with a perforated ulcer in the first part of the duodenum which was managed by falciform ligament patch instead of the usual omental patch. In cases of a deficient or unhealthy greater omentum, or if it cannot be brought in the upper part of the abdominal cavity due to severe adhesions, the falciform ligament can be used efficiently in the closure of perforated duodenal ulcer.

## INTRODUCTION

Perforated duodenal ulcers are one of the most serious healthcare concerns with a high mortality rate among ineffectively managed patients. Different surgical modalities either open or laparoscopic have been proposed for the closure of the perforated ulcer. The use of an omental patch is considered the standard of management. However, in some select patients where the greater omentum is either unviable, unhealthy, or cannot be utilized, the falciform ligament can be used as an adequate patch for closure.

## CASE PRESENTATION

**Figure 1:**
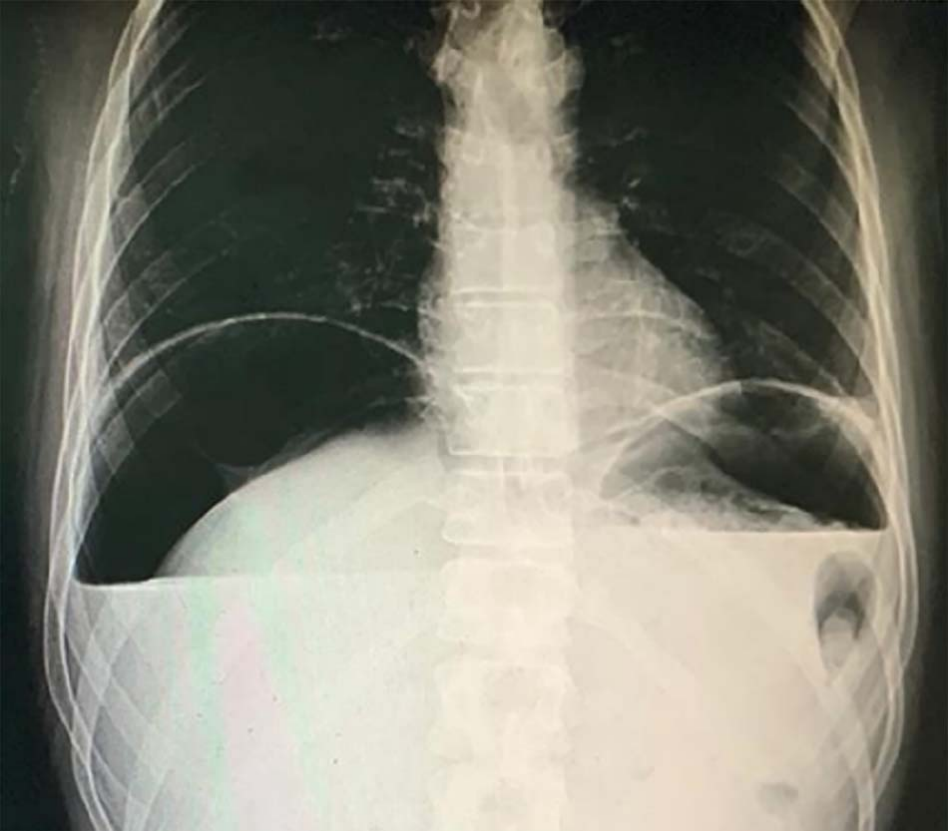
Chest X-ray showing air under the diaphragm.

A 38-year-old male intravenous drug user presented to the emergency room with right iliac fossa pain of 3-hour duration. He had a previous surgical history of laparotomy and pan-proctocolectomy with ileoanal anastomosis indicated for his familial polyposis coli. Examination showed severe epigastric and right iliac fossa tenderness and rebound tenderness with a marked abdominal rigidity. Blood results showed blood urea nitrogen, 84; creatinine, 1.1; hematocrit, 63; K, 2.8; Na, 142; and total leukocytic count, 16.1. A chest X-ray was done and showed free air under the diaphragm (Fig. 1). An ultrasound showed a marked pelvic, peri splenic and perihepatic turbid collection.

**Figure 2:**
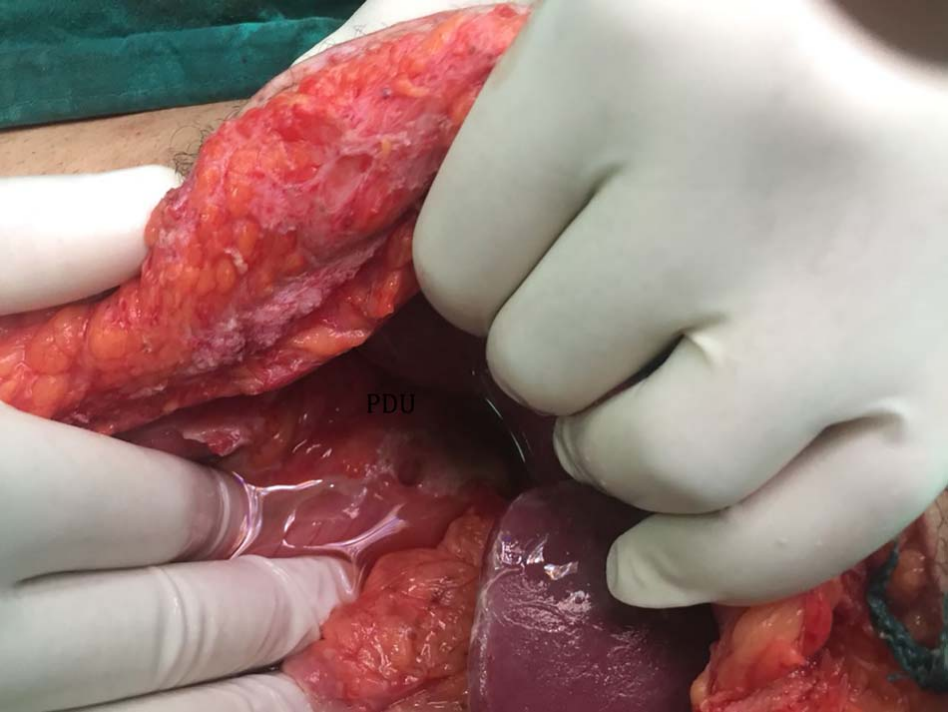
2-cm ulcer appeared on the anterior aspect of the first part of the duodenum.

**Figure 3:**
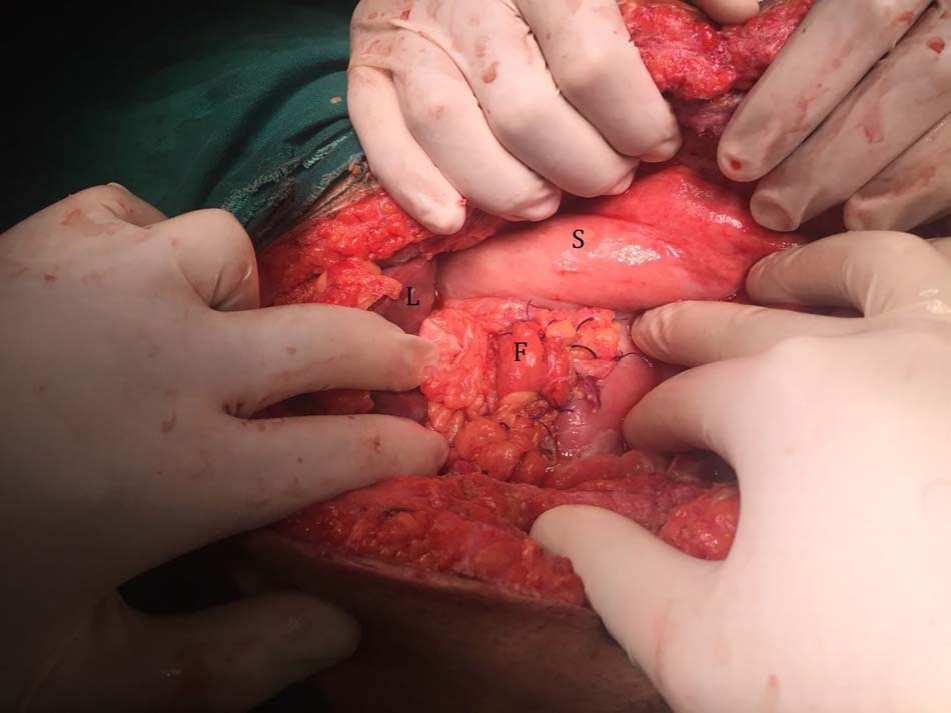
Duodenal ulcer after repair using falciform ligament.

After resuscitation with 2 liters of crystalloids in the form of 1-L 0.9% sodium chloride and 1-L Hartmann’s solution-Ringer acetate, the patient was transferred to the operation room, and a midline abdominal laparotomy was performed. After the evacuation of about 2 liters of collection from the abdominal cavity and peritoneal lavage done, a 2-cm ulcer defect appeared on the anterior aspect of the first part of duodenum (Fig. 2). There was no healthy omentum left from the previous operation to provide an adequate omental patch for repair. Therefore the falciform ligament was released and used as a patch for the ulcer that was fixed to cover the defect using interrupted Vicryl 2/0 sutures (Fig. 3). Methylene blue stain injection test was done through a nasogastric tube and no leakage was found. The postoperative course was uneventful, and contrast study was done on the seventh day postoperatively showing no contrast leakage and the patient was discharged.

The patient came for follow-up 1 week after discharge and was well. Abdominal examination showed a lax, non-tender abdomen that is freely movable with respiration, and repeat ultrasound was normal. His wound showed infection with serous discharge which was managed by daily dressing, and finally the patient was instructed to have follow-up upper GI endoscopy after 3 months.

## DISCUSSION

The incidence and prevalence rates of duodenal ulcers have markedly decreased worldwide owed mainly to *H. pylori* eradication which is considered the main causative factor that leads to duodenal ulcers [1–3].

However, overall peptic ulcer disease complications are more common hemorrhages than perforations. Duodenal ulcer perforations stem to be the most feared complication overall and a commonly encountered surgical emergency. Despite recent advances in ulcer treatment, mortality rates as high as 10–40% have been reported without even an immediate diagnosis and management [4–6].

Different surgical modalities have been proposed for the emergency management of perforated duodenal ulcer like excision of the whole perforated part of the duodenum, jejunal serosal patch closure and duodenostomy for some selected cases, but the widely used ones are Graham’s omentopexy and its modification. In this technique several interrupted sutures are taken through the defect untied; then the greater omentum is brought between these sutures and finally tying the sutures to hold the omentum in place. And for the success of this technique, the omentum should be viable and not strangulated [7, 8].

In Graham’s omentopexy, interrupted sutures are taken through the ulcer defect -without attaching- utilizing absorbable threads such as Vicryl 2/0 to bring the vascularized omental flap between these sutures and the sutures held over it [7].

In the modification of Graham’s omentopexy, the defect is firstly closed by the sutures and holding them then the omentum is brought over the closed defect, and after that tying the primary suture over it again adding more strength to the repair [8].

Fry *et al*. were the first to use the falciform ligament after adequate mobilization in the repair of perforated peptic ulcer [9].

The middle segment artery of the liver and the left phrenic artery give the main blood supply of the falciform ligament making it a well-vascularized structure to be used as a flap [10].

Munro *et al*. use the falciform ligament in the repair of perforated duodenal ulcers in six patients; they describe the ease of its utilization, especially in laparoscopic surgery [11].

In the setting of previous several operations in which there is unhealthy or deficient omentum, the falciform ligament could be used in the repair of perforated duodenal ulcer after liberation from the anterior abdominal wall. Several sutures are taken in the ulcer defect like that of Graham repair and then placing the falciform ligament in between these sutures with subsequent tying of sutures over it. In our case the falciform ligament was used efficiently to seal the perforated ulcer in first part of the duodenum with no intraoperative or postoperative leakage, and the patient was discharged after full recovery without any postoperative complications or eventful follow-ups.

In conclusion, the falciform ligament can be used efficiently in the closure of perforated duodenal ulcer especially in patients with deficient greater omentum or in cases in which the greater omentum cannot be brought in the upper part of the abdominal cavity due to severe adhesions.

## CONFLICT OF INTEREST STATEMENT

The authors have no conflict of interest.

## FUNDING

None.
